# Regulation of Brown and White Adipocyte Transcriptome by the Transcriptional Coactivator NT-PGC-1α

**DOI:** 10.1371/journal.pone.0159990

**Published:** 2016-07-25

**Authors:** Jihyun Kim, Vivian E. Fernand, Tara M. Henagan, Jeho Shin, Peter Huypens, Susan Newman, Thomas W. Gettys, Ji Suk Chang

**Affiliations:** 1 Laboratory of Gene Regulation and Metabolism, Pennington Biomedical Research Center, Baton Rouge, LA, United States of America; 2 Laboratory of Nutrient Sensing and Adipocyte Signaling, Pennington Biomedical Research Center, Baton Rouge, LA, United States of America; 3 Genomics Core, Pennington Biomedical Research Center, Baton Rouge, LA, United States of America; Northeast Ohio Medical University, UNITED STATES

## Abstract

The β_3_-adrenergic receptor (AR) signaling pathway is a major component of adaptive thermogenesis in brown and white adipose tissue during cold acclimation. The β_3_-AR signaling highly induces the expression of transcriptional coactivator PGC-1α and its splice variant N-terminal (NT)-PGC-1α, which in turn activate the transcription program of adaptive thermogenesis by co-activating a number of transcription factors. We previously reported that NT-PGC-1α is able to increase mitochondrial number and activity in cultured brown adipocytes by promoting the expression of mitochondrial and thermogenic genes. In the present study, we performed genome-wide profiling of NT-PGC-1α-responsive genes in brown adipocytes to identify genes potentially regulated by NT-PGC-1α. Canonical pathway analysis revealed that a number of genes upregulated by NT-PGC-1α are highly enriched in mitochondrial pathways including fatty acid transport and β-oxidation, TCA cycle and electron transport system, thus reinforcing the crucial role of NT-PGC-1α in the enhancement of mitochondrial function. Moreover, canonical pathway analysis of NT-PGC-1α-responsive genes identified several metabolic pathways including glycolysis and fatty acid synthesis. In order to validate the identified genes *in vivo*, we utilized the FL-PGC-1α^-/-^ mouse that is deficient in full-length PGC-1α (FL-PGC-1α) but expresses a slightly shorter and functionally equivalent form of NT-PGC-1α (NT-PGC-1α^254^). The β_3_-AR-induced increase of NT-PGC-1α^254^ in FL-PGC-1α^-/-^ brown and white adipose tissue was closely associated with elevated expression of genes involved in thermogenesis, mitochondrial oxidative metabolism, glycolysis and fatty acid synthesis. Increased adipose tissue thermogenesis by β_3_-AR activation resulted in attenuation of adipose tissue expansion in FL-PGC-1α^-/-^ adipose tissue under the high-fat diet condition. Together, the data strengthen our previous findings that NT-PGC-1α regulates mitochondrial genes involved in thermogenesis and oxidative metabolism in brown and white adipocytes and further suggest that NT-PGC-1α regulates a broad spectrum of genes to meet cellular needs for adaptive thermogenesis.

## Introduction

Brown adipose tissue (BAT) is specialized for energy expenditure via UCP1-mediated thermogenesis as a defense against hypothermia and obesity [1–3]. Recently, adult humans have been shown to possess functional BAT whose activity is increased by cold exposure [4–6]. In response to cold, norepinephrine released from the sympathetic nerve endings stimulates β-adrenergic receptors (β-ARs) on BAT [7]. In particular, a β_3_-AR signaling pathway plays a crucial role in BAT thermogenesis, leading to a marked increase in triacylglycerol mobilization, mitochondrial fatty acid oxidation and UCP1 expression/activity [2, 8, 9]. In rodents, prolonged stimulation of β_3_-AR in white adipose tissue induces emergence of brown-like (beige) adipocytes, which have been suggested to serve to increase energy dissipation through enhanced mitochondrial oxidative metabolism and UCP1-mediated thermogenesis [10–12].

The transcriptional coactivator PGC-1α is a master regulator of adaptive thermogenesis. During cold stress, PGC-1α is highly induced by cold-stimulated β_3_-AR signaling in brown and brown-like (beige) adipocytes and activates a number of nuclear receptors and transcription factors for transcriptional induction of UCP1 and other mitochondrial genes involved in mitochondrial oxidative metabolism [9, 13]. Previously, we reported that alternative splicing of the PGC-1α gene produces a splice variant encoding the N-terminal isoform of PGC-1α (NT-PGC-1α) that lacks the 271–797 amino acids of full-length PGC-1α (FL-PGC-1α) [14]. NT-PGC-1α is co-expressed with FL-PGC-1α, and its mRNA and protein levels are largely elevated by cold-stimulated β_3_-AR/cAMP signaling in brown adipocytes [14]. NT-PGC-1α is a functional transcriptional coactivator containing the transcription activation and nuclear receptor interaction domains of FL-PGC-1α [14, 15]. Accordingly, expression of NT-PGC-1α in cultured brown adipocytes significantly induces the expression of UCP1 and mitochondrial genes, leading to an increase in mitochondrial content and respiratory activity [14–16]. Moreover, NT-PGC-1α alone is sufficient to activate cold-induced BAT thermogenesis in FL-PGC-1α^-/-^ mice by promoting the expression of thermogenic and mitochondrial genes in the absence of FL-PGC-1α [14, 15, 17, 18]. In contrast, ablation of both FL-PGC-1α and NT-PGC-1α impairs BAT thermogenesis, leading to a complete inability of PGC-1α^-/-^ mice to defend against cold stress [19].

NT-PGC-1α interacts with various nuclear receptors through the nuclear receptor interaction motif (LxxLL) [14, 15]. Nuclear receptors regulate a large number of genes involved in mitochondrial biogenesis and oxidative metabolism, thermogenesis, glycolysis, and lipid metabolism [20]. Thus, this raises the possibility that NT-PGC-1α regulate a broad range of genes involved in several metabolic pathways in brown adipocytes. Genome-wide analysis of NT-PGC-1α-regulated genes has not been conducted to date. We therefore aimed to identify genes that are potentially regulated by NT-PGC-1α in brown adipocytes using microarray analysis. The identified candidate genes were subsequently validated using quantitative real-time PCR in NT-PGC-1α-expressing PGC-1α^-/-^ brown adipocytes and 3T3-L1 white adipocytes. In order to include genes with physiological significance, the candidate genes were further validated in FL-PGC-1α^-/-^ mice that have genetic ablation of FL-PGC-1α but retain the expression of a slightly shorter and functionally equivalent form of NT-PGC-1α (NT-PGC-1α^254^) [15, 21]. NT-PGC-1α^254^ is a functional transcriptional coactivator containing 251 amino acids of NT-PGC-1α (aa 1–270), followed by 3 additional amino acids created by the duplicated exon 3 [21]. We here identify a set of NT-PGC-1α-regulated genes whose expression is correlated with cold/β_3_-AR-induced BAT thermogenesis.

## Materials and Methods

### Animal Experiments

All animal handling and experiments were conducted according to the procedures reviewed and approved by the Pennington Biomedical Research Center Institutional Animal Care and Use Committee (PBRC IACUC). All mice were housed on a 12-h light/12-h dark cycle. Mice were sacrificed with CO_2_ inhalation_,_ followed by cervical dislocation. The present animal study was approved by the PBRC IACUC in the protocols 659 (March 23, 2010) and 740 (August 22, 2011). Brown adipose tissue was dissected from the interscapular region and inguinal white adipose tissue was dissected from the layer under the skin in the region of the groin. Tissue was snap-frozen in liquid nitrogen. FL-PGC-1α^-/-^ mice deficient in FL-PGC-1α have been described previously [15, 16, 21]. The PGC-1α gene product produced in FL-PGC-1α^-/-^ mice was defined as NT-PGC-1α^254^ to distinguish it from naturally occurring NT-PGC-1α. *Cohort1*: 9 to 10-week-old FL-PGC-1α^-/-^ male mice and wild-type controls (n = 8 per group) were singly housed and provided a high-fat (45 kcal % fat) diet (D12451, Research Diets, Inc., New Brunswick, NJ) *ad libitum* for 2 weeks. Thereafter, mice were weighed and their body composition was determined by NMR using a Bruker Mouse Minispec (Bruker Optics, Billerica, MA) prior to transfer of the mice into indirect calorimetry chambers. After a 24h acclimation period, each mouse was monitored for 6 days for determination of VO_2_ and VCO_2_ using a Comprehensive Laboratory Animal Monitoring System (Columbus Instruments, Columbus, OH). Locomotor activity was measured using an OPTO-M3 sensor system while the mice were in the chamber. After 3 days in the chamber, mice were switched to the same high-fat diet but formulated to contain 0.001% CL316243 as described previously [15, 22], and oxygen consumption was continuously monitored for 4 additional days. Thereafter, mice removed from the chambers were assessed for body weight and composition and provided the high-fat diet containing 0.001% CL316243 for 2 additional days prior to harvest of brown and white adipose tissue. Energy expenditure was calculated as (VO2 × [3.815 + (1.232 × RQ)] × 4.187) and expressed as kilojoules per hour per lean mass for analysis. In parallel, age-matched WT and FL-PGC-1α^-/-^ mice (n = 8 per group) fed a high-fat (45 kcal % fat) diet for 3 weeks were used as controls.

*Cohort 2*: For cold exposure experiments, 9 to 10-week-old WT and FL-PGC-1α^-/-^ mice (n = 7–9 per group) fed a high-fat (45 kcal % fat) diet for 3 weeks at 28°C were singly housed and exposed to 4°C for 5 h. Core rectal temperature was measured at baseline and every 1 h over the 5 h-period as described previously [15].

### Microarray Experiment

Immortalized brown preadipocytes expressing hemagglutinin-tagged NT-PGC-1α-HA or empty vector [14] were differentiated as described previously [14]. Total RNA was prepared using TriReagent (Invitrogen Life Technologies, Inc., Carlsbad, CA) and the RNeasy RNA purification protocol (QIAGEN, Valencia, CA). RNA quantity and quality were measured using the RNA 6000 Nano LabChip kit with the Agilent 2100 Biolanalyzer (Agilent Technologies, Palo Alto, CA). Three RNA samples were pooled for each group. Microarray analysis was performed in the Genomics Core of the Pennington Biomedical Research Center. Briefly, one microgram of RNA was amplified and labeled using the NanoAmp IVT-Amplification Kit according to the manufacturer’s protocol (Applied Biosystems, Foster City, CA). For each array comparison, ten micrograms of digoxigenin-11-UTP-labeled (Roche Diagnostics, Indianapolis, IN) cRNA was fragmented using the Applied Biosystems Chemiluminescent Detection Kit protocol. Pooled samples were hybridized onto ABI Mouse Genome Survey arrays, with approximately 32,000 genes and 1,000 controls represented, for 16 h in a rotating hybridization oven, followed by washing and application of the chemiluminescent substrate, according to the Applied Biosystems Chemiluminescent Detection Kit protocol (Applied Biosystems, Foster City, CA). The microarray slides were scanned and gridded, and the images were digitized and analyzed using an Applied Biosystems 1700 Chemiluminescent Microarray Analyzer, along with the package ABarray v1.36.0 (Applied Biosystems, Foster City, CA). Data files were analyzed using the statistical program R v.2.2.1 (http://www.r-project.org) and the quantile-quantile method for normalization across arrays (http://www.bioconductor.org). Parameters for feature control were set at signal/noise ratio ≥3 and quality flags <5,000 in order to generate fold changes for features. The raw and normalized datasets were deposited to the National Center for Biotechnology Information (NCBI)’s Gene Expression Omnibus (GEO) database (accession number GSE71774).

### Pathway Analysis

Ingenuity Pathway Analysis (IPA) software (http://www.ingenuity.com) was used for functional analysis of differentially expressed genes by NT-PGC-1α and their networks. Only genes with a *p* value smaller than 0.05 and a fold change greater than 1.25 (NT-PGC-1α versus empty vector) were selected from the normalized data, uploaded into the Ingenuity software and mapped to the gene object in the Ingenuity Pathways Knowledge Base for pathway analysis. A *p* value less than 0.05 indicates that the association between a set of focus genes in microarray and a given pathway is statistically significant, non-random association. The right-tailed Fisher Exact Test was used to calculate a *p* value. Metabolic pathways and signal transduction pathways (AMPK signaling, PPARα/RXRα activation, RAR activation and p38 MAPK activation) were selected for analysis, and genes that are involved in top-ranked canonical pathways were displayed by overlapping the involved metabolic and signal transduction pathways.

### Cellular Oxygen Consumption Assay

Immortalized brown preadipocytes expressing NT-PGC-1α-HA or lentiviral vector were seeded at a density of 40,000 cells per well in XF24 Cell Culture microplates and differentiated as described previously [14]. Before starting the experiment, cells were washed and allowed for equilibration in Krebs Henseleit Buffer (KHB) containing 2.5 mM glucose for 20–30 minutes at 37°C. The cell plates were then placed into the Seahorse Bioscience XF24 instrument and oxygen consumption was measured in real time. After 3 measurements of basal oxygen consumption, a β-AR agonist isoproterenol (10 μM) was injected into the wells and subsequent oxygen consumption was measured 5 more times at 6.5 min intervals.

### Quantitative Real-Time PCR Analysis

PGC-1α^-/-^ brown preadipocytes were transduced with retrovirus expressing pBABE retroviral empty vector, NT-PGC-1α-a-HA and NT-PGC-1α-b-HA. Similarly, 3T3-L1 preadipocytes were transduced with retrovirus expressing pBABE or NT-PGC-1α-a-HA. Brown and white preadipocytes were induced for differentiation as described previously [17, 23]. Total RNA from adipose tissue and adipocytes was reverse-transcribed for quantitative real-time PCR analysis as described previously [15, 17]. Relative mRNA expression of the genes of interest was determined after normalization to cyclophilin by standard curve and ΔΔCt methods. For quantitative assessment of mitochondrial biogenesis, the ratio of mitochondrial to nuclear DNA was analyzed using quantitative RT-PCR with primers for the NADH dehydrogenase subunit 1 (ND1) and lipoprotein lipase (LPL) as described previously [15].

### Western Blot Analysis

Whole cell extracts were prepared from tissues and adipocytes by homogenization in lysis buffer [17] and subjected to Western blot analysis using following antibodies: anti-PGC-1α [14], anti-UCP1 [14, 24], anti-α-tubulin (Abcam) and anti-β-actin (Sigma). Protein concentration was determined using Bio-Rad DC protein assay reagents according to the manufacturer’s instructions.

### Statistical Analysis

Data are presented as mean ± SD. Student *t* test or ANOVA was used to compare the difference between groups using Graphad Prism 5 software. Values of *P* < 0.05 were considered statistically significant.

## Results

### Genome-Wide Gene Expression Profiling of NT-PGC-1α-Responsive Genes in Brown Adipocytes

**Fig 1:**
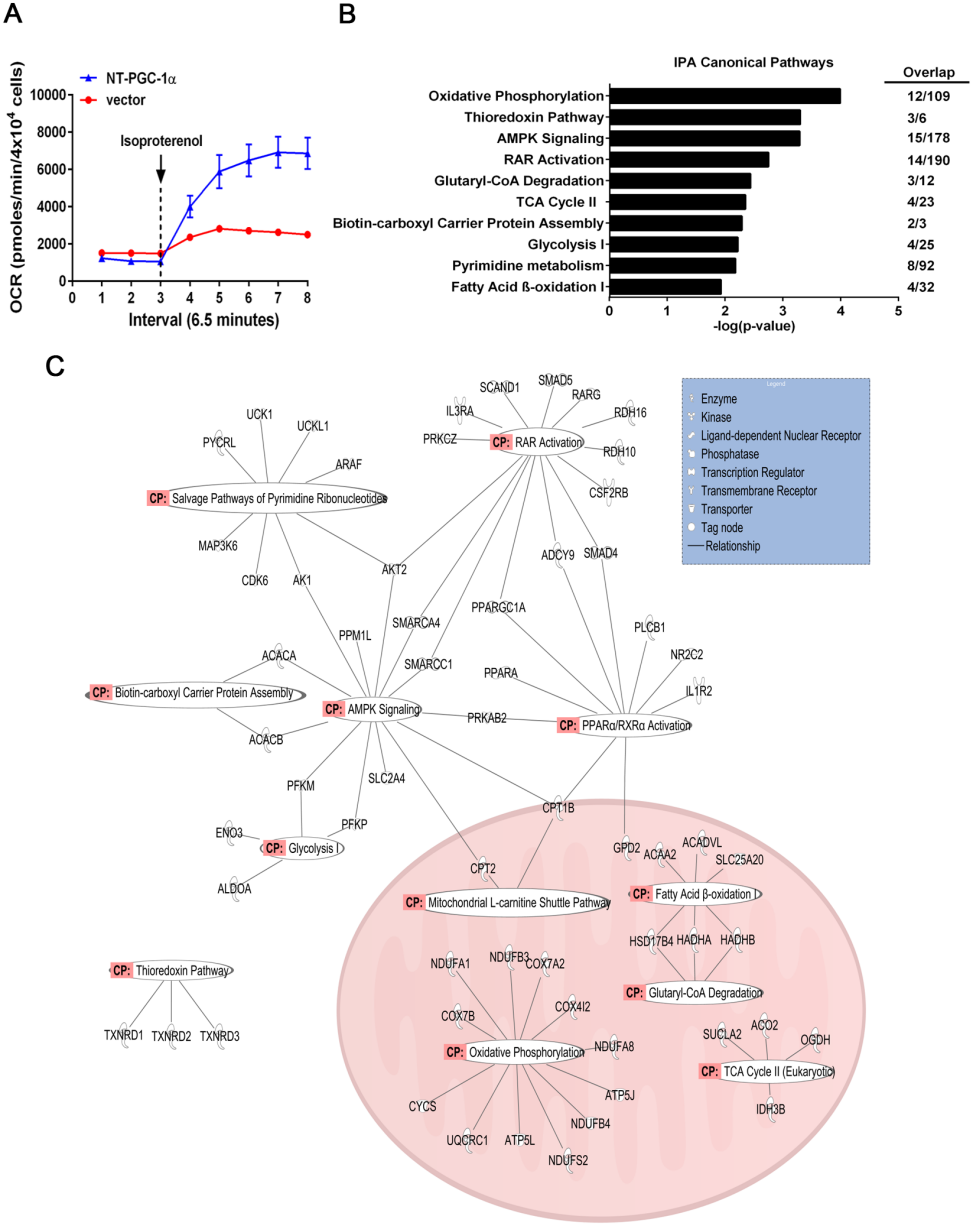
Pathway analysis of NT-PGC-1α-responsive genes in brown adipocytes. (A) Oxygen consumption rates of brown adipocytes expressing NT-PGC-1α or empty vector. Basal and isoproterenol-stimulated mitochondrial respiration (n = 5) was measured as described in Materials and Methods. (B) Ingenuity pathway analysis on the genes whose expression was elevated by NT-PGC-1α compared to controls in brown adipocytes. The statistically significant canonical pathways identified are listed according to their –log (*p*-value). The number of overlapping genes detected in each pathway was shown. (C) Functional interaction analysis of 65 genes that are enriched in the metabolic and signal transduction pathways identified by the IPA analysis. Genes or gene products are displayed as nodes that represent the functional class of the gene product, and a biological relationship between two genes is represented as a line.

We previously showed that expression of NT-PGC-1α in brown adipocytes increases mitochondrial biogenesis and UCP1 gene expression [14]. In agreement with these findings, NT-PGC-1α-expressing brown adipocytes exhibited higher rates of oxygen consumption compared to control cells in response to β-adrenergic receptor agonist isoproterenol (Fig 1A), indicating that NT-PGC-1α-mediated mitochondrial gene expression increases mitochondrial respiratory capacity. To identify the set of genes potentially regulated by NT-PGC-1α in brown adipocytes, we carried out microarray analysis on brown adipocytes expressing NT-PGC-1α or empty vector. Expression levels of NT-PGC-1α in brown adipocytes were 7-fold higher compared to control samples (S1 Table) and this increase is comparable to the cold-induced levels of NT-PGC-1α mRNA in brown adipose tissue (BAT) [14, 15]. The microarray data were initially filtered to contain only genes up- or down-regulated at least 1.5-fold by NT-PGC-1α with a *p* value smaller than 0.05. The analysis yielded 1023 genes, of which 446 genes were upregulated and 577 genes were downregulated by NT-PGC-1α compared with empty vector control (S1 Table). Among 446 genes, many mitochondrial genes (e.g. Cox4i2, Cox5b, Atp5l, Ndufa8, Ndufb3, Ndufc2, Uqcrc1) were filtered out because they were induced less than 1.5-fold in NT-PGC-1α microarray data (S1 Table). Despite the small increase in mitochondrial gene expression, NT-PGC-1α overexpression leads to increased mitochondrial content and respiratory function [14]. Thus, the genes whose expression was increased by NT-PGC-1α with a fold change greater than 1.25 (765 genes) were selected for further analysis (S1 Table).

Canonical pathway analysis using the Ingenuity Pathways Analysis (IPA) software identified several pathways that appear regulated by NT-PGC-1α, including mitochondrial oxidative phosphorylation, thioredoxin pathway, AMPK signaling, RAR activation, glutaryl-CoA degradation, TCA cycle, fatty acid synthesis, glycolysis, pyrimidine metabolism and fatty acid β-oxidation (Fig 1B and 1C). Many pathways identified in Fig 1 were also preserved when analyzed with a set of genes with FC > 1.5 and *p* < 0.05 (S2 Table).

The main effect of NT-PGC-1α overexpression on brown adipocyte transcriptome was upregulation of genes involved in mitochondrial metabolism, such as acetyl-CoA production via fatty acid β-oxidation and glutaryl-CoA degradation, TCA cycle, and respiratory electron transport system. Among 1097 mouse MitoCarta genes collected with a high confidence [25], NT-PGC-1α upregulated 195 mitochondrial genes that consist of core mitochondrial components including TCA cycle, electron transport chain subunits, mitochondrial protein import machinery, mitochondrial carriers and mitochondrial ribosomal proteins (S3 Table). We previously reported that NT-PGC-1α highly elevates the expression of mitochondrial UCP1 by being recruited to the UCP1 enhancer region (-2442 to -2770) in cAMP-treated brown adipocytes [14]. However, UCP1 gene expression was not significantly elevated in NT-PGC-1α-microarray probably due to the culture condition lacking cAMP signaling.

Identification of genes involved in AMPK, RAR and PPARα/RXRα signaling pathways is interesting given that AMPK activity is increased during cold acclimation in brown and white adipose tissue [26] and that RAR and PPARα/RXRα play an important role in the regulation of UCP1 expression and lipid metabolism in brown adipocytes [27–30]. Upregulation of genes involved in glycolytic and lipogenic pathways is in agreement with previous findings that glucose uptake/metabolism, fatty acid mobilization and synthesis are increased as part of the adaptive thermogenic processes in brown adipose tissue during cold acclimation [3, 31–34].

### NT-PGC-1α Regulates a Set of Genes Involved in Mitochondrial Oxidative Metabolism, Glycolysis and Fatty Acid Synthesis in Brown and White Adipocytes

**Fig 2:**
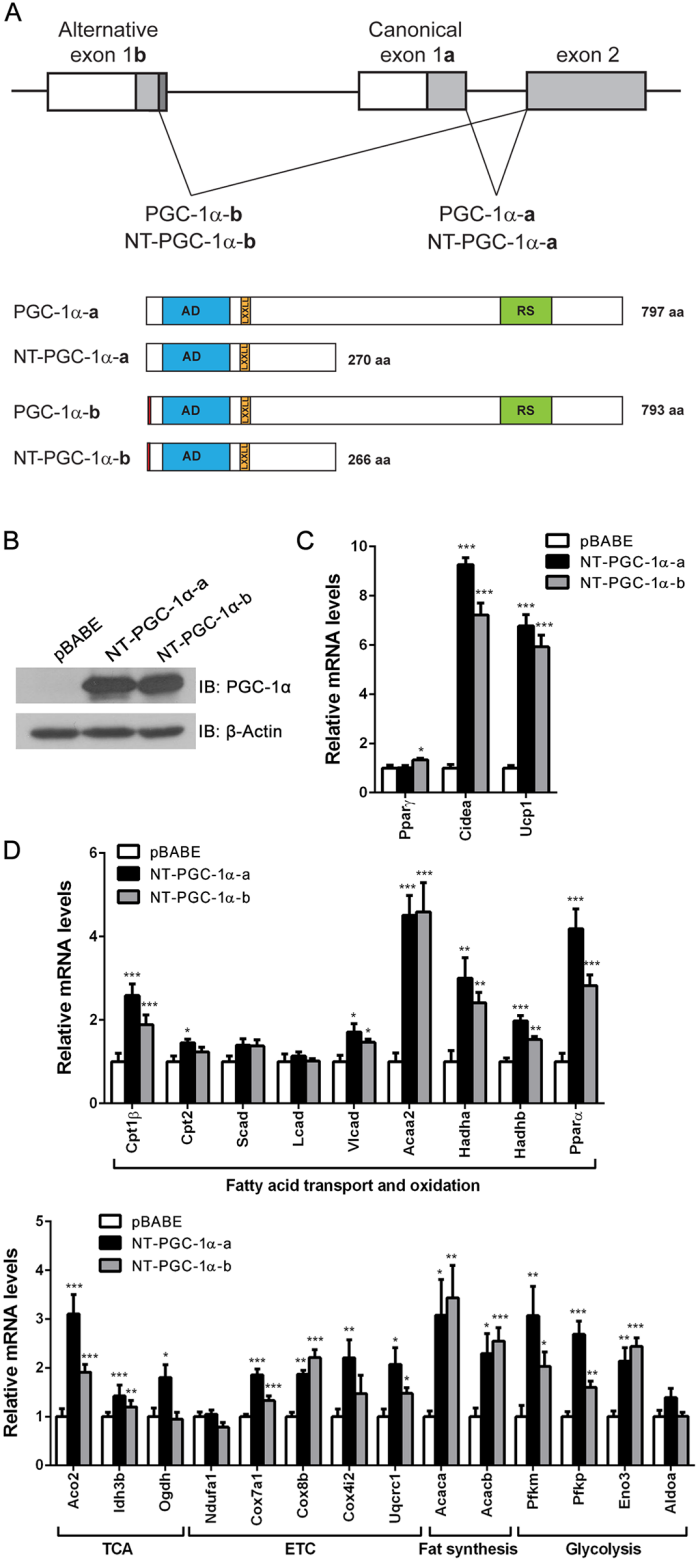
The effect of NT-PGC-1α on candidate gene expression in brown adipocytes. (A) Schematic diagram of splicing events of the PGC-1α gene. Splicing between canonical exon 1a and exon 2 produces PGC-1α-a and NT-PGC-1α-a, whereas alternative splicing between distal exon 1b and exon 2 produces PGC-1α-b and NT-PGC-1α-b. AD, transcription activation domain; LxxLL, nuclear receptor interaction motif; RS, arginine/serine-rich domain. (B) Expression of NT-PGC-1α-a and NT-PGC-1α-b proteins in PGC-1α-null brown adipocytes. (C) Quantitative real-time PCR analysis of gene expression in PGC-1α-null brown adipocytes expressing pBABE empty vector, NT-PGC-1α-a or NT-PGC-1α-b (n = 8). Empty vector vs NT-PGC-1α-a or NT-PGC-1α-b: *P < 0.05, **P < 0.01, ***P < 0.001.

Given that mitochondrial oxidative metabolism, glycolysis and fatty acid synthesis are increased during adaptive thermogenesis in BAT, we chose for validation a set of genes identified in these metabolic pathways including oxidative phosphorylation, fatty acid β-oxidation, glutaryl-CoA degradation, TCA cycle, glycolysis and fatty acid synthesis (Fig 1). To validate the candidate genes, real-time PCR analysis was carried out in PGC-1α^-/-^ brown adipocytes expressing NT-PGC-1α or empty vector. Overexpression of NT-PGC-1α induced the expression of known target genes, CIDEA and UCP1, in PGC-1α^-/-^ brown adipocytes (Fig 2C, black bars). Genes involved in fatty acid transport and oxidation (Cptlβ, Cpt2, Vlcad, Acaa2, Hadha, Hadhb, PPARα), TCA cycle (Aco2, Idh3b, Ogdh), electron transport chains (Cox7a1, Cox8b, Cox4i2, Uqcrc1), fatty acid synthesis (Acaca, Acacb) and glycolysis (Pfkm, Pfkp, Eno3) were significantly upregulated by NT-PGC-1α (Fig 2D, black bars).

Previously, our group and others reported that PGC-1α-b and NT-PGC-1α-b (also reported as PGC-1α4 [35]) are expressed from the alternative exon 1b that is located 13.7kb upstream of the canonical exon 1a of the PGC-1α gene [15, 36]. PGC-1α-b and NT-PGC-1α-b are 4 amino acids shorter than canonical PGC-1α-a and NT-PGC-1α-a, respectively, with 12 amino acid differences at the N-terminus (Fig 2A). While PGC-1α-a and NT-PGC-1α-a are dominant isoforms at basal conditions, PGC-1α-b and NT-PGC-1α-b are highly induced by cold in BAT [15]. NT-PGC-1α-b is a functional transcriptional coactivator containing the transcription activation and nuclear receptor interaction domains of NT-PGC-1α-a [15]. Accordingly, we overexpressed NT-PGC-1α-b in PGC-1α^-/-^ brown adipocytes (Fig 2B) and directly compared the effects of NT-PGC-1α-a and NT-PGC-1α-b on candidate gene expression. Overexpression of NT-PGC-1α-b comparably induced the expression of CIDEA and UCP1 in PGC-1α^-/-^ brown adipocytes (Fig 2C). Likewise, a number of candidate genes induced by NT-PGC-1α-a were also highly induced by NT-PGC-1α-b in similar fashion (Fig 2D), demonstrating that NT-PGC-1α-a and NT-PGC-1α-b are functionally comparable in regulating gene expression in brown adipocytes.

**Fig 3:**
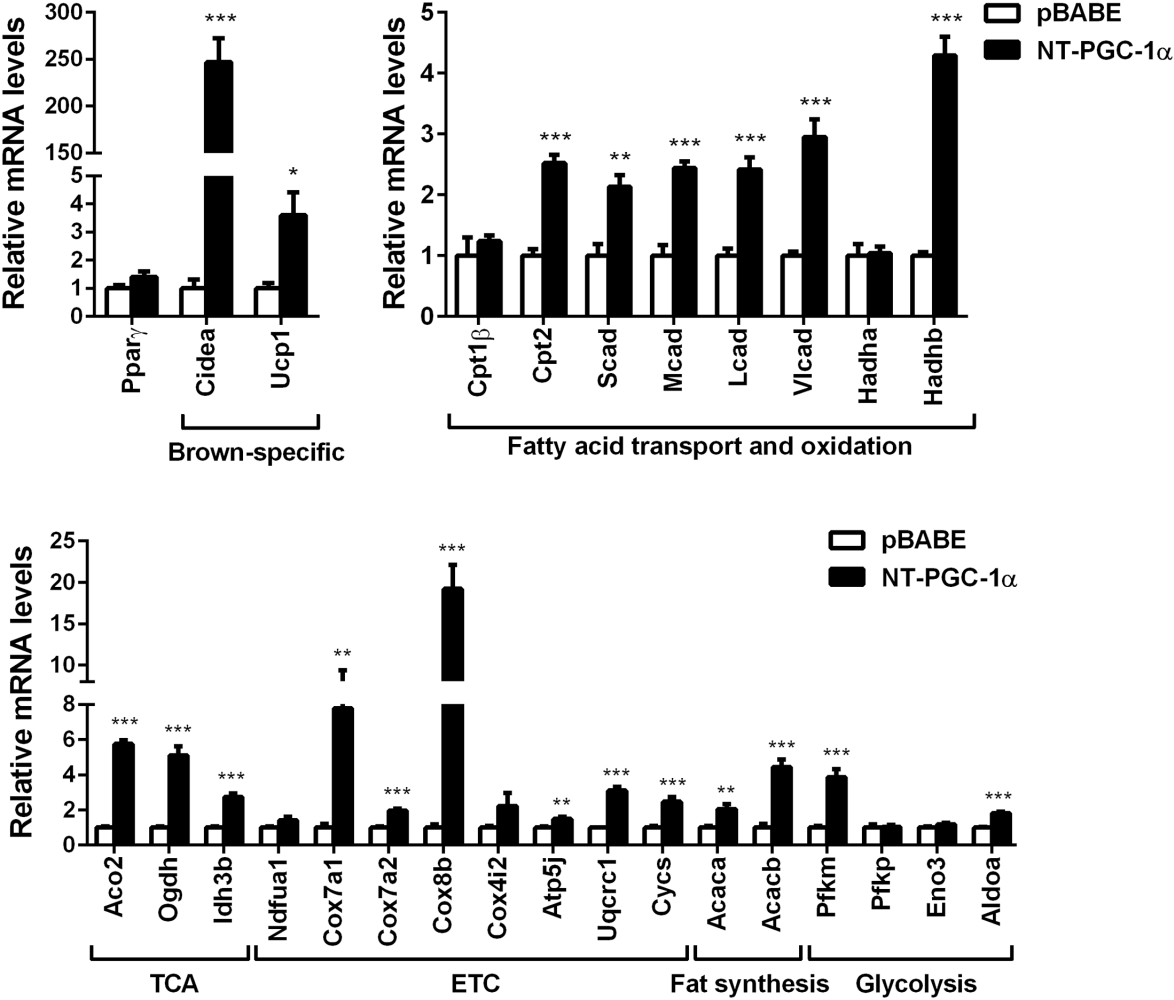
The effect of NT-PGC-1α on candidate gene expression in 3T3-L1 adipocytes. Quantitative real-time PCR analysis of gene expression in 7-day-differentiated 3T3-L1 adipocytes expressing pBABE empty vector or NT-PGC-1α-a (n = 6). Empty vector vs NT-PGC-1α: *P < 0.05, **P < 0.01, ***P < 0.001.

Next, we examined whether a set of NT-PGC-1α-responsive genes validated in brown adipocytes are also regulated by NT-PGC-1α in 3T3-L1 white adipocytes. NT-PGC-1α strongly induced the expression of brown adipocyte-specific genes, CIDEA and UCP1 (Fig 3). Moreover, expression of mitochondrial fatty acid oxidation (Cpt2, Scad, Mcad, Lcad, Vlcad, Hadhb), TCA cycle (Aco2, Ogdh, Idh3b), ETC (Cox7a1, Cox7a2, Cox8b, Atp5j, Uqcrc1, Cycs), lipogenic (Acaca, Acacb) and glycolic (Pfkm, Aldoa) genes were significantly elevated by NT-PGC-1α in 3T3-L1 white adipocytes (Fig 3).

### Effects of NT-PGC-1α^254^ Activation by β_3_-Adrenergic Stimulation on Adipose Tissue Transcriptome in FL-PGC-1α^-/-^ Mice

The qPCR analyses of NT-PGC-1α-responsive genes in brown and white adipocytes show that NT-PGC-1α regulates a number of metabolic genes involved in thermogenesis, mitochondrial oxidative metabolism, fatty acid synthesis and glycolysis.

**Fig 4:**
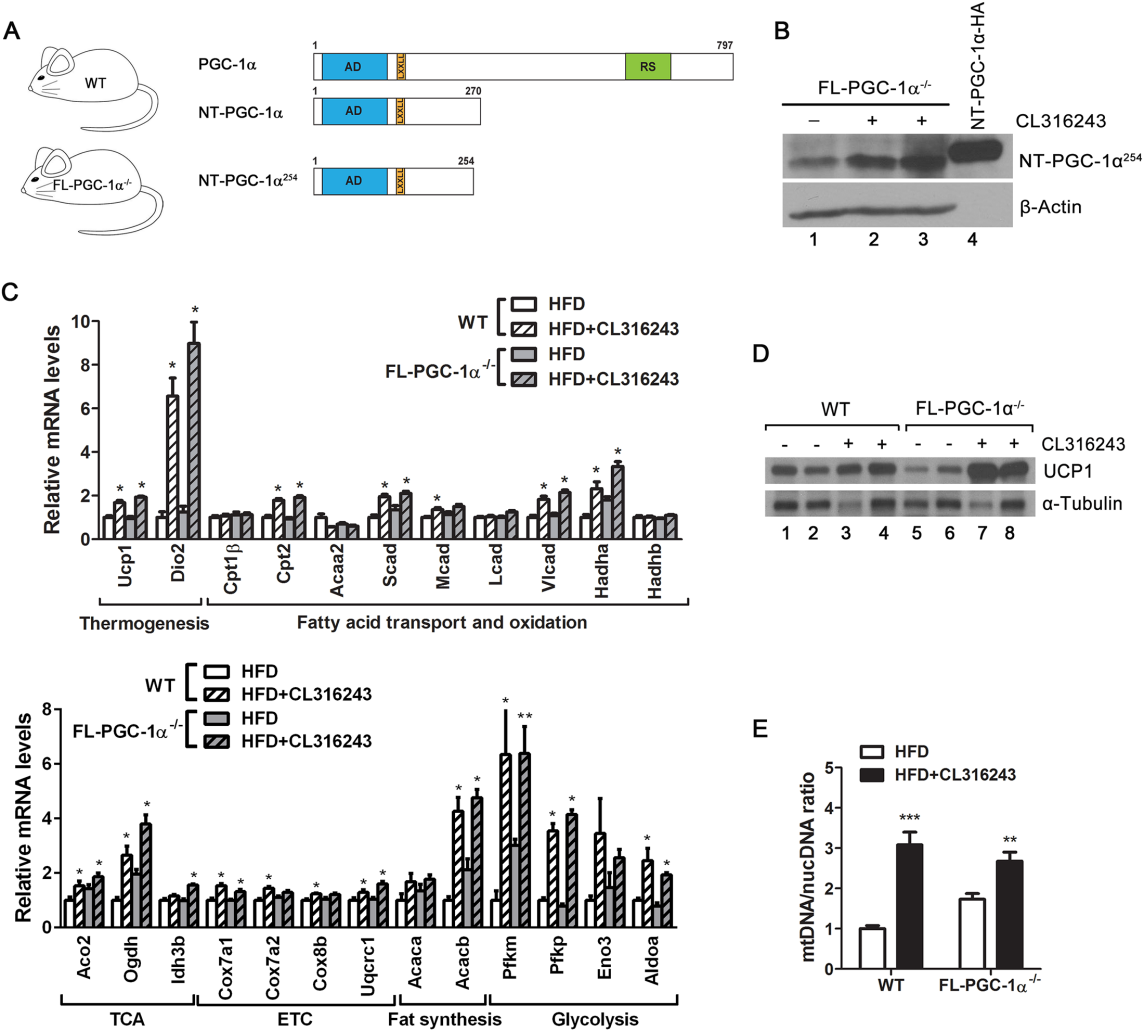
Gene expression changes in FL-PGC-1α^-/-^ brown adipose tissue in response to CL316243. (A) Schematic diagram of WT mice expressing PGC-1α and NT-PGC-1α and of FL-PGC-1α^-/-^ mice only expressing NT-PGC-1α^254^. AD, transcription activation domain; LxxLL, nuclear receptor interaction motif; RS, arginine/serine-rich domain. At the age of 9 to 10 weeks, WT and FL-PGC-1α^-/-^ mice (n = 8 per group) were singly housed and provided a high-fat diet (HFD) *ad libitum* for 2 weeks, followed by the same diet containing 0.001% CL316243 (HFD + CL316243) for 6 days. (B) Increased expression of NT-PGC-1α^254^ by β_3_-AR activation in FL-PGC-1α^-/-^ BAT. NT-PGC-1α-HA in HEK293 cells was used as a positive control. (C) Quantitative real-time PCR analysis of a number of metabolic genes in WT and FL-PGC-1α^-/-^ BAT (n = 7–8 per group). HFD vs HFD+CL316243: *P < 0.05, **P < 0.01. (D) Protein levels of UCP1 in BAT whole cell extracts. Identical amounts of proteins were loaded, and α-tubulin was used as a loading control. (E) Quantitative analysis of mitochondrial biogenesis. The ratio of mitochondrial DNA (mtDNA) relative to nuclear genome (nucDNA) was analyzed in BAT (n = 7–8 per group). **P < 0.01, ***P < 0.001.

In order to further validate the *in vitro* gene expression data *in vivo*, we utilized FL-PGC-1α^-/-^ mice that are deficient in full-length PGC-1α (FL-PGC-1α) but retain the expression of a slightly shorter but functionally equivalent form of NT-PGC-1α (NT-PGC-1α^254^) (Fig 4A). NT-PGC-1α^254^ preserves the transcription activation (AD) and nuclear receptor interaction (LxxLL) domains of NT-PGC-1α, thus having the same ability to promote gene expression in brown adipocytes by activating various nuclear receptors [15, 16]. To induce endogenous NT-PGC-1α^254^ expression in brown and white adipose tissue, FL-PGC-1α^-/-^ mice were treated with a highly selective β_3_-AR agonist, CL316243, for 6 days. Stimulation of β_3_-AR in brown and white adipose tissue activates cAMP signaling pathways, which subsequently activate the PGC-1α gene promoter [9, 13].

Expression of NT-PGC-1α^254^ was elevated by β_3_-AR activation in FL-PGC-1α^-/-^ BAT (Fig 4B, lanes 2–3). The increase in NT-PGC-1α^254^ protein levels was correlated with increased expression of its target gene Ucp1 in CL316243-stimulated FL-PGC-1α^-/-^ BAT (Fig 4C). We previously demonstrated that cold-induced NT-PGC-1α^254^ is recruited to the UCP1 enhancer region (-2442 to -2770), contributing to increased expression of UCP1 in response to cold [15]. Consistent with elevated mRNA levels, UCP1 protein levels were higher in CL316243-stimulated BAT compared to controls in both genotypes (Fig 4D, lanes 3–4 and 7–8). Moreover, CL316243-induced increase in NT-PGC-1α^254^ protein was closely associated with CL316243-induced increase in expression of mitochondrial fatty acid oxidation (Cpt2, Scad, Vlcad, Hadha), TCA cycle (Aco2, Ogdh, Idh3b) and electron transport chain genes (Cox7a1, Uqcrc1) in FL-PGC-1α^-/-^ BAT (Fig 4C). The ratio of mitochondrial DNA relative to nuclear genome was increased by β_3_-AR activation in WT and FL-PGC-1α^-/-^ BAT, indicating increased mitochondrial biogenesis in both genotypes (Fig 4E). In addition, expression of lipogenic (Acacb) and glycolic genes (Pfkm, Pfkp, Aldoa) was elevated in CL316243-stimulated WT and FL-PGC-1α^-/-^ BAT compared to controls (Fig 4C). These data suggest that NT-PGC-1α^254^ regulates a set of genes involved in thermogenesis, mitochondrial oxidative metabolism, fatty acid synthesis and glycolysis in the absence of FL-PGC-1α in BAT.

**Fig 5:**
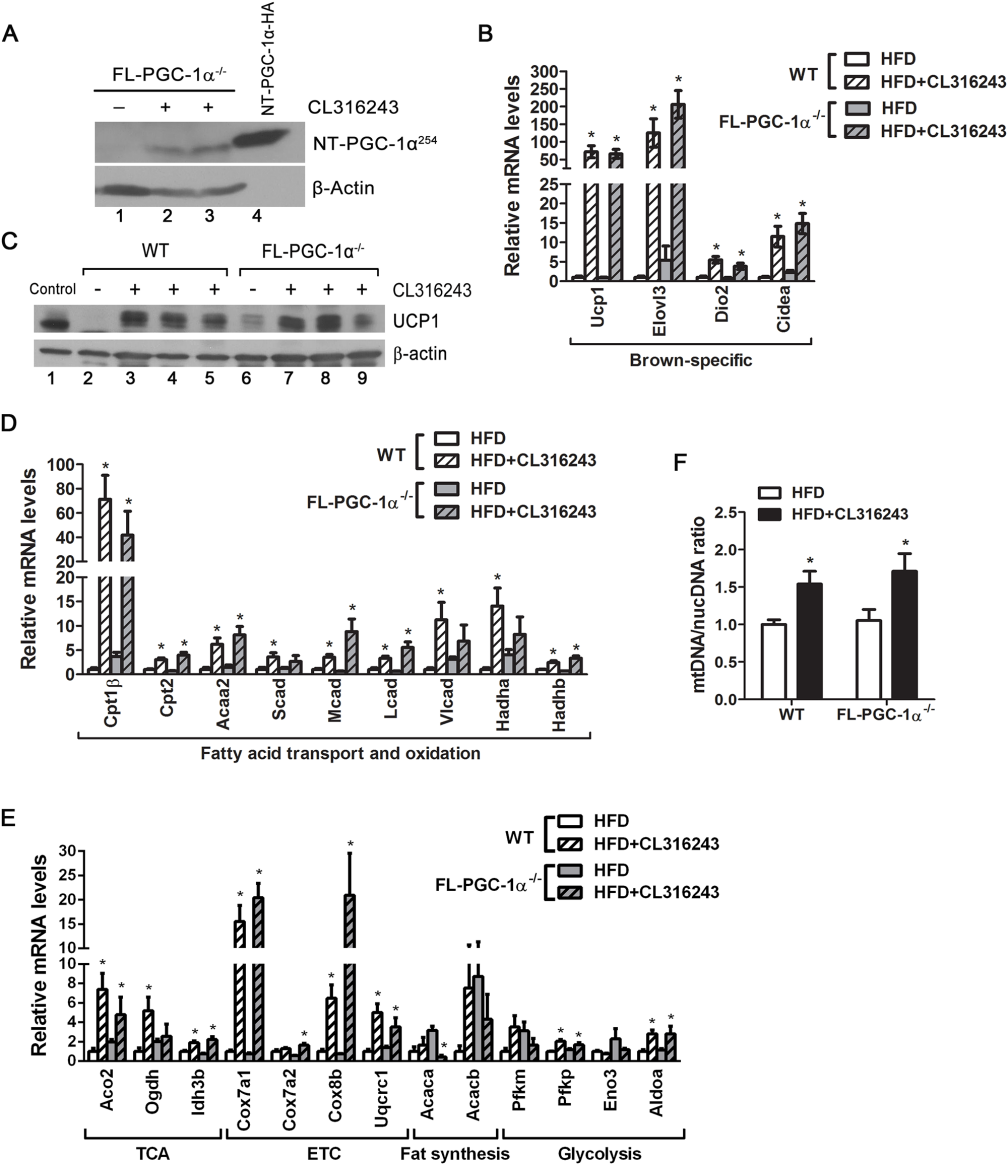
Gene expression changes in FL-PGC-1α^-/-^ inguinal white adipose tissue in response to CL316243. (A) Increased expression of NT-PGC-1α^254^ by β_3_-AR activation in FL-PGC-1α^-/-^ IWAT. NT-PGC-1α-HA in HEK293 cells was used as a positive control. FL-PGC-1α^-/-^ mice were fed a HFD for 2 weeks, followed by the treatment with or without CL316243 for 6 days on HFD. (B, D, E) Quantitative real-time PCR analysis of a number of metabolic genes in WT and FL-PGC-1α^-/-^ IWAT (n = 8 per group). HFD vs HFD+CL316243: *P < 0.05. (C) Western blot analysis of UCP1 expression in IWAT whole cell extracts (100 μg). BAT extracts (10 μg) were added to WT IWAT extract (- CL316243) as a positive control for UCP1. (F) The ratio of mitochondrial DNA (mtDNA) relative to nuclear genome (nucDNA) was analyzed in IWAT (n = 8 per group). *P < 0.05.

Prolonged stimulation of β_3_-AR in inguinal white adipose tissue (IWAT) induces emergence of brown-like (beige) adipocytes, which increase the thermogenic capacity of white adipose tissue by enhanced mitochondrial oxidative metabolism and UCP1-mediated thermogenesis [10–12]. Stimulation of β_3_-AR markedly induced NT-PGC-1α^254^ expression in IWAT (Fig 5A, lanes 2–3). The increase of NT-PGC-1α^254^ protein by CL316243 was associated with increased expression of known target genes, Ucp1 and Cidea, in CL316243-stimulated FL-PGC-1α^-/-^ IWAT compared to controls (Fig 5B). The increase in Ucp1 gene expression was in agreement with a large increase in UCP1 protein levels in CL316243-stimulated FL-PGC-1α^-/-^ IWAT (Fig 5C). Other brown adipocyte-specific genes such as Elovl3 and Dio2 were strongly induced by β_3_-AR activation in WT and FL-PGC-1α^-/-^ IWAT, indicating comparable levels of browning in both IWAT (Fig 5B). The increase in NT-PGC-1α^254^ protein by CL316243 was closely associated with increased expression of a set of mitochondrial genes involved in fatty acid oxidation, TCA cycle and electron transport system in CL316243-stimulated FL-PGC-1α^-/-^ IWAT compared to controls (Fig 5D and 5E). The mitochondrial content was also elevated in CL316243-treated groups as evidenced by the increased ratio of mitochondrial DNA normalized to nuclear genome (Fig 5F).

### Gene Expression Changes in FL-PGC-1α^-/-^ Adipose Tissue in Response to CL316243 Are Accompanied by Increased Adipose Tissue Thermogenesis

**Fig 6:**
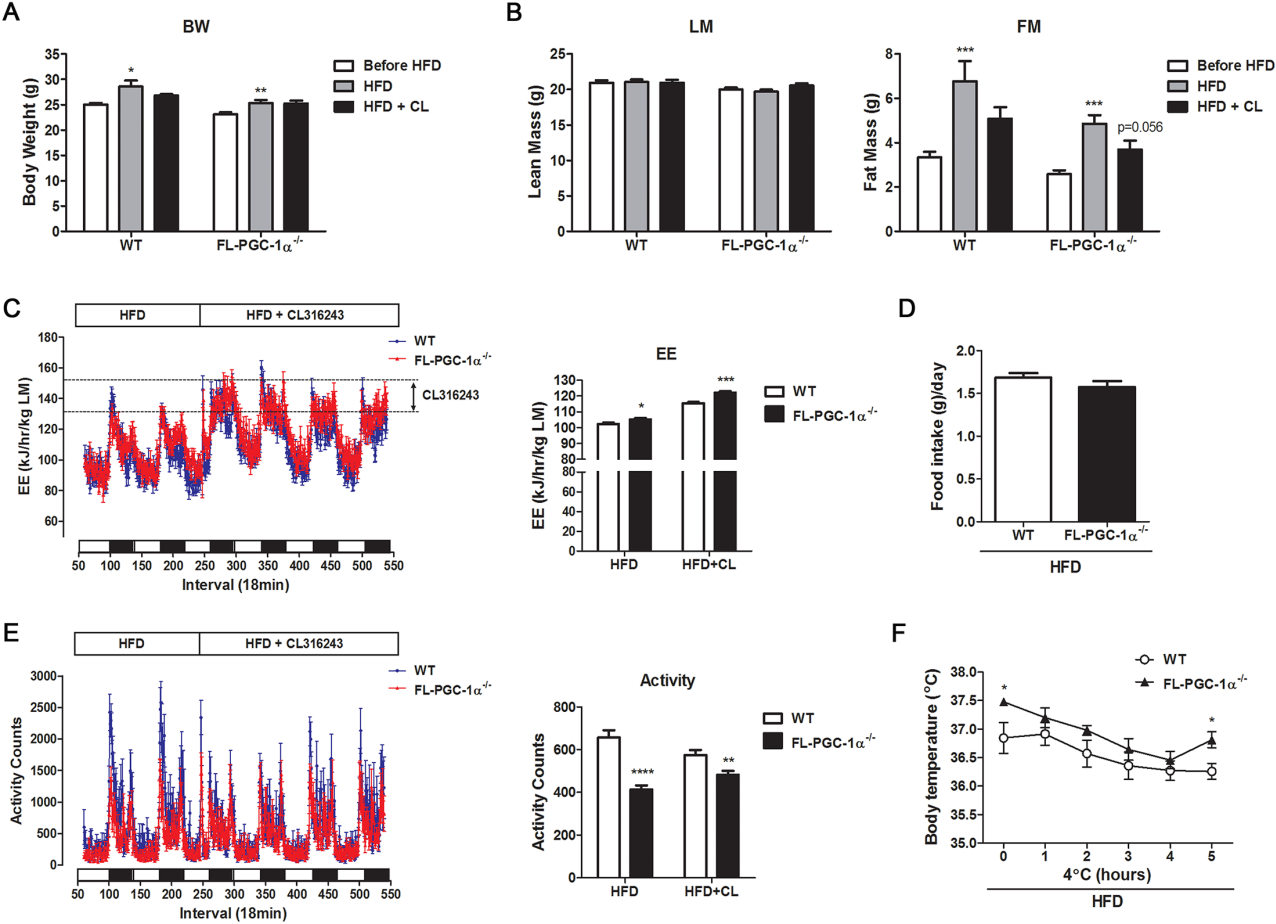
An increase in adipose tissue-driven thermogenesis in FL-PGC-1α^-/-^ mice attenuates adipose tissue expansion under the HFD. (A, B) Body weight and composition of WT and FL-PGC-1α^-/-^ mice. Mice were measured for body weight and composition prior to introduction of HFD, after 2 weeks on HFD and after additional 6 days on CL 316243+HFD. BW: body weight, LM: lean mass, FM: fat mass (C) Energy expenditure (EE) of WT and FL-PGC-1α^-/-^ mice. Mice were measured for VO_2_ and VCO_2_ on HFD for first two days and on HFD + CL316243 for additional four days. EE (kilojoules per hour) was calculated as described in Materials and Methods and expressed per lean mass (LM). (D) Average food intake of WT and FL-PGC-1α^-/-^ mice on HFD. Food intake was expressed as grams per day per mouse. (E) Voluntary activity of WT and FL-PGC-1α^-/-^ mice. Ambulatory activity was monitored at 18 min intervals for 6 days on HFD and HFD + CL316243. (F) Core body temperature of WT and FL-PGC-1α^-/-^ mice (n = 7–9 per group). Mice fed a HFD for 3 weeks were exposed to 4°C and their core body temperature was measured for 5h using a MicroTherma thermometer with the RET-3 mouse rectal probe. *P < 0.05, **P < 0.01, ***P < 0.001, ****P < 0.0001.

Activation of β_3_-AR in FL-PGC-1α^-/-^ brown and white adipose tissue induced adaptive changes in gene expression. We next investigated the effect of β_3_-AR activation on adipose tissue thermogenesis in FL-PGC-1α^-/-^ mice. FL-PGC-1α^-/-^ mice were fed with a high-fat diet (HFD) for 2 weeks prior to treatment with CL316243 and assessed for body weight and body composition before and after CL316243 treatment for 6 days. WT and FL-PGC-1α^-/-^ mice exhibited significant weight gain after 2 weeks on HFD (Fig 6A). The observed increase in body weight was mainly due to an increase in fat mass in WT and FL-PGC-1α^-/-^ mice (Fig 6B). Food intake did not differ in both genotypes (Fig 6D). Activation of adipose tissue by CL316243 for 6 days prevented HDF-induced adipose tissue expansion in WT and FL-PGC-1α^-/-^ mice (Fig 6B).

Next, we measured whole body energy expenditure (EE) in each mouse prior to and after administration of CL316243 by monitoring O_2_ consumption and CO_2_ production using indirect calorimetry. Given that β_3_-AR expression is limited to adipose tissue [37, 38], the increase in whole body EE after administration of CL316243 accounts for the energy expenditure mediated by adipose tissue thermogenesis. CL316243 immediately increased EE in HFD-fed WT and FL-PGC-1α^-/-^ mice and produced continuous increases in the EE during a 4-day measurement. WT and FL-PGC-1α^-/-^ mice had little or no alteration in locomotor activity by CL316243 (Fig 6E).We also evaluated the thermogenic capacity of HFD-fed FL-PGC-1α^-/-^ mice under cold stress. WT and FL-PGC-1α^-/-^ mice were able to maintain body temperature over the 5 h-period of cold exposure at 4°C (Fig 6F). Collectively, these results indicate that gene expression changes in FL-PGC-1α^-/-^ brown and white adipose tissue by β_3_-AR activation are accompanied by increased adipose tissue thermogenesis and attenuation of HFD-induced adipose tissue expansion.

## Discussion

Transcriptional coactivator NT-PGC-1α is produced by alternative splicing of the PGC-1α gene. NT-PGC-1α lacks the 271–797 amino acids of full-length PGC-1α (FL-PGC-1α) but retains the transcriptional activation and nuclear receptor interaction domains of FL-PGC-1α [14]. NT-PGC-1α regulates many PGC-1α target genes including UCP1 and mitochondrial genes, leading to increased mitochondrial content and respiration in brown adipocytes [14, 16–18]. Genome-wide gene expression profiling of NT-PGC-1α-responsive genes in brown adipocytes revealed that the main effect of NT-PGC-1α overexpression on the brown adipocyte transcriptome was the enrichment in the expression of genes involved in mitochondrial metabolism, such as fatty acid β-oxidation, TCA cycle and respiratory electron transport system. Maximal thermogenesis by UCP1 has a strong dependence on fatty acid availability and β-oxidation and subsequent fluxes through the TCA cycle and electron transport system to generate an electrochemical proton gradient across the inner mitochondrial membrane. In agreement with the microarray data, NT-PGC-1α-expressing brown adipocytes showed increased mitochondrial respiration compared to control cells. NT-PGC-1α overexpression in 3T3-L1 white adipocytes also strongly induced the expression of UCP1 and a number of mitochondrial genes involved in fatty acid β-oxidation, TCA cycle and respiratory electron transport system, indicating that NT-PGC-1α regulates white adipocyte transcriptome.

IPA analysis of genes whose expression is increased by NT-PGC-1α further identified additional metabolic pathways including glycolysis, fatty acid synthesis, and pyrimidine metabolism. Identification of genes involved in glycolysis and lipogenesis is in agreement with previous findings that glucose uptake/metabolism, fatty acid mobilization and synthesis are increased as part of the adaptive thermogenic processes in brown adipose tissue during cold acclimation [3, 31–34]. Glycerol-3-phosphate produced from glucose serves as the backbone of triacylglycerol for fatty acid re-esterification [31]. NT-PGC-1α-dependent regulation of genes involved in these metabolic pathways suggests that NT-PGC-1α may influence lipid droplet replenishment as well as oxidation of fatty acids for thermogenesis. Upregulation of genes involved in pyrimidine metabolism may be related to increased transcription and cellular metabolism given that pyrimidine nucleotides are nucleic acid subunits and carriers of activated intermediates. Further study will be required to elucidate whether changes in gene expression by NT-PGC-1α lead to changes in glycolysis and lipogenesis in brown and white adipocytes.

The microarray analysis showed that 577 genes are downregulated at least 1.5-fold by NT-PGC-1α in brown adipocytes. Gene products upregulated by NT-PGC-1α may indirectly repress transcription of these genes in brown adipocytes. IPA analysis of 577 genes identified several pathways including acute phase response signaling, VDR/RXR signaling, and IL-12 signaling (S4 Table).

PGC-1α and NT-PGC-1α are co-expressed in brown adipocytes. Many genes and metabolic pathways identified by NT-PGC-1α microarray analysis are overlapping with those regulated by PGC-1α [39]. PGC-1α and NT-PGC-1α may regulate common and distinct sets of genes in the same metabolic pathway for maximal gene expression in response to cold [17]. The expression patterns of thermogenic, mitochondrial and metabolic genes are varied during the course of cold exposure [31]. For example, a subset of genes is rapidly induced in response to cold with an early trend upward (5h), whereas others are gradually induced during 24-48h of cold exposure. Time course analysis of PGC-1α and NT-PGC-1α expression revealed that while the protein levels of PGC-1α were maximal at 5h exposure to cold and rapidly degraded, NT-PGC-1α protein was gradually increased with highest expression at the 1 day of cold exposure and relatively stable throughout the cold exposure (our unpublished data). Further study will be required to elucidate whether there is difference in the timing of their action in cold-induced gene expression in brown adipose tissue.

Ablation of PGC-1α and NT-PGC-1α in PGC-1α^-/-^ mice attenuates cold/β_3_-AR-induced expression of UCP1 in brown adipose tissue [19] and leads to reduced expression of UCP1 in inguinal white adipose tissue [40, 41]. The present study showed that β_3_-AR-induced increase of NT-PGC-1α^254^ was closely associated with increased expression of UCP1 in FL-PGC-1α^-/-^ brown and white adipose tissue. We previously demonstrated that cold-induced NT-PGC-1α^254^ is recruited to the UCP1 enhancer region in FL-PGC-1α^-/-^ BAT for UCP1 gene expression [15]. Thus, we think that NT-PGC-1α^254^, which has the same ability to coactivate various nuclear receptors [15, 16], promotes the expression of UCP1 and a number of mitochondrial and metabolic genes in brown adipocytes in response to β_3_-AR activation. However, we cannot rule out the possibility that other factors contribute to the observed increase in mitochondrial and metabolic gene expression as well.

Collectively, the present study strengthens our previous findings that NT-PGC-1α plays a crucial role in mitochondrial oxidative metabolism and thermogenesis and further suggests that it regulates a broad spectrum of thermogenic processes in brown and white adipocytes.

## Supporting Information

S1 Table A list of genes differentially regulated by NT-PGC-1α in brown adipocytes. (XLSX)

S2 Table Pathway analysis of genes upregulated by NT-PGC-1α in brown adipocytes. Ingenuity pathway analysis was performed on genes upregulated by NT-PGC-1α (FC > 1.5 and *p* < 0.05).
(XLS)

S3 Table A list of MitoCarta genes identified in NT-PGC-1α microarray. Analysis was performed on genes upregulated by NT-PGC-1α (FC > 1.25 and *p* < 0.05).
(XLSX)

S4 Table Pathway analysis of genes downregulated by NT-PGC-1α in brown adipocytes. Ingenuity pathway analysis was performed on genes downregulated by NT-PGC-1α (FC > 1.5 and *p* < 0.05).
(XLS)

## References

[1] Nedergaard J, Golozoubova V, Matthias A, Asadi A, Jacobsson A, Cannon B. UCP1: the only protein able to mediate adaptive non-shivering thermogenesis and metabolic inefficiency. Biochim Biophys Acta. 2001;1504(1):82–106. Epub 2001/03/10. S0005-2728(00)00247-4 [pii]. . 11239487 10.1016/s0005-2728(00)00247-4

[2] Golozoubova V, Hohtola E, Matthias A, Jacobsson A, Cannon B, Nedergaard J. Only UCP1 can mediate adaptive nonshivering thermogenesis in the cold. FASEB J. 2001;15(11):2048–2050. Epub 2001/08/21. 10.1096/fj.00-0536fje 00-0536fje [pii]. . 11511509

[3] Cannon B, Nedergaard J. Brown adipose tissue: function and physiological significance. Physiol Rev. 2004;84(1):277–359. Epub 2004/01/13. 10.1152/physrev.00015.2003 84/1/277 [pii]. . 14715917

[4] Cypess AM, Lehman S, Williams G, Tal I, Rodman D, Goldfine AB, et al Identification and importance of brown adipose tissue in adult humans. N Engl J Med. 2009;360(15):1509–1517. Epub 2009/04/10. 10.1056/NEJMoa0810780 360/15/1509 [pii]. 19357406 PMC2859951

[5] Virtanen KA, Lidell ME, Orava J, Heglind M, Westergren R, Niemi T, et al Functional brown adipose tissue in healthy adults. N Engl J Med. 2009;360(15):1518–1525. Epub 2009/04/10. 10.1056/NEJMoa0808949 360/15/1518 [pii]. . 19357407

[6] van der Lans AA, Hoeks J, Brans B, Vijgen GH, Visser MG, Vosselman MJ, et al Cold acclimation recruits human brown fat and increases nonshivering thermogenesis. J Clin Invest. 2013;123(8):3395–3403. Epub 2013/07/23. 10.1172/JCI68993 68993 [pii]. 23867626 PMC3726172

[7] Landsberg L, Saville ME, Young JB. Sympathoadrenal system and regulation of thermogenesis. Am J Physiol. 1984;247(2 Pt 1):E181–189. Epub 1984/08/01. . 6380306 10.1152/ajpendo.1984.247.2.E181

[8] Langin D, Ekholm D, Ridderstrale M, Lafontan M, Belfrage P. cAMP-dependent protein kinase activation mediated by beta 3-adrenergic receptors parallels lipolysis in rat adipocytes. Biochim Biophys Acta. 1992;1135(3):349–352. Epub 1992/06/29. 0167-4889(92)90242-4 [pii]. . 1352466 10.1016/0167-4889(92)90242-4

[9] Wu Z, Puigserver P, Andersson U, Zhang C, Adelmant G, Mootha V, et al Mechanisms controlling mitochondrial biogenesis and respiration through the thermogenic coactivator PGC-1. Cell. 1999;98(1):115–124. . 10412986 10.1016/S0092-8674(00)80611-X

[10] Cousin B, Cinti S, Morroni M, Raimbault S, Ricquier D, Penicaud L, et al Occurrence of brown adipocytes in rat white adipose tissue: molecular and morphological characterization. J Cell Sci. 1992;103 (Pt 4):931–942. Epub 1992/12/01. . 1362571 10.1242/jcs.103.4.931

[11] Guerra C, Koza RA, Yamashita H, Walsh K, Kozak LP. Emergence of brown adipocytes in white fat in mice is under genetic control. Effects on body weight and adiposity. J Clin Invest. 1998;102(2):412–420. Epub 1998/07/17. 10.1172/JCI3155 9664083 PMC508900

[12] Granneman JG, Li P, Zhu Z, Lu Y. Metabolic and cellular plasticity in white adipose tissue I: effects of beta3-adrenergic receptor activation. Am J Physiol Endocrinol Metab. 2005;289(4):E608–616. Epub 2005/06/09. 00009.2005 [pii] 10.1152/ajpendo.00009.2005 . 15941787

[13] Puigserver P, Wu Z, Park CW, Graves R, Wright M, Spiegelman BM. A cold-inducible coactivator of nuclear receptors linked to adaptive thermogenesis. Cell. 1998;92(6):829–839. . 9529258 10.1016/s0092-8674(00)81410-5

[14] Zhang Y, Huypens P, Adamson AW, Chang JS, Henagan TM, Lenard NR, et al Alternative mRNA splicing produces a novel biologically active short isoform of PGC-1{alpha}. J Biol Chem. 2009;284(47):32813–32826. 10.1074/jbc.M109.037556 19773550 PMC2781698

[15] Chang JS, Fernand V, Zhang Y, Shin J, Jun HJ, Joshi Y, et al NT-PGC-1alpha protein is sufficient to link beta3-adrenergic receptor activation to transcriptional and physiological components of adaptive thermogenesis. J Biol Chem. 2012;287(12):9100–9111. Epub 2012/01/28. M111.320200 [pii] 10.1074/jbc.M111.320200 22282499 PMC3308807

[16] Jun HJ, Joshi Y, Patil Y, Noland RC, Chang JS. NT-PGC-1alpha activation attenuates high-fat diet-induced obesity by enhancing brown fat thermogenesis and adipose tissue oxidative metabolism. Diabetes. 2014;63(11):3615–3625. Epub 2014/05/23. 10.2337/db13-1837 db13-1837 [pii]. 24848065 PMC4207386

[17] Chang JS, Huypens P, Zhang Y, Black C, Kralli A, Gettys TW. Regulation of NT-PGC-1alpha subcellular localization and function by protein kinase A-dependent modulation of nuclear export by CRM1. J Biol Chem. 2010;285(23):18039–18050. Epub 2010/03/31. M109.083121 [pii] 10.1074/jbc.M109.083121 20351112 PMC2878565

[18] Jun HJ, Gettys TW, Chang JS. Transcriptional Activity of PGC-1alpha and NT-PGC-1alpha Is Differentially Regulated by Twist-1 in Brown Fat Metabolism. PPAR Res. 2012;2012:320454 Epub 2012/10/25. 10.1155/2012/320454 23093952 PMC3474972

[19] Lin J, Wu PH, Tarr PT, Lindenberg KS, St-Pierre J, Zhang CY, et al Defects in adaptive energy metabolism with CNS-linked hyperactivity in PGC-1alpha null mice. Cell. 2004;119(1):121–135. . 15454086 10.1016/j.cell.2004.09.013

[20] Francis GA, Fayard E, Picard F, Auwerx J. Nuclear receptors and the control of metabolism. Annu Rev Physiol. 2003;65:261–311. Epub 2003/01/09. 10.1146/annurev.physiol.65.092101.142528 092101.142528 [pii]. . 12518001

[21] Leone TC, Lehman JJ, Finck BN, Schaeffer PJ, Wende AR, Boudina S, et al PGC-1alpha deficiency causes multi-system energy metabolic derangements: muscle dysfunction, abnormal weight control and hepatic steatosis. PLoS Biol. 2005;3(4):e101 Epub 2005/03/12. 04-PLBI-RA-0782R2 [pii] 10.1371/journal.pbio.0030101 15760270 PMC1064854

[22] Surwit RS, Feinglos MN, Rodin J, Sutherland A, Petro AE, Opara EC, et al Differential effects of fat and sucrose on the development of obesity and diabetes in C57BL/6J and A/J mice. Metabolism. 1995;44(5):645–651. Epub 1995/05/01. 0026-0495(95)90123-X [pii]. . 7752914 10.1016/0026-0495(95)90123-x

[23] Floyd ZE, Stephens JM. Interferon-gamma-mediated activation and ubiquitin-proteasome-dependent degradation of PPARgamma in adipocytes. J Biol Chem. 2002;277(6):4062–4068. Epub 2001/12/26. 10.1074/jbc.M108473200 M108473200 [pii]. . 11733495

[24] Commins SP, Watson PM, Padgett MA, Dudley A, Argyropoulos G, Gettys TW. Induction of uncoupling protein expression in brown and white adipose tissue by leptin. Endocrinology. 1999;140(1):292–300. Epub 1999/01/14. 10.1210/endo.140.1.6399 . 9886838

[25] Pagliarini DJ, Calvo SE, Chang B, Sheth SA, Vafai SB, Ong SE, et al A mitochondrial protein compendium elucidates complex I disease biology. Cell. 2008;134(1):112–123. Epub 2008/07/11. 10.1016/j.cell.2008.06.016 S0092-8674(08)00768-X [pii]. 18614015 PMC2778844

[26] Mulligan JD, Gonzalez AA, Stewart AM, Carey HV, Saupe KW. Upregulation of AMPK during cold exposure occurs via distinct mechanisms in brown and white adipose tissue of the mouse. J Physiol. 2007;580(Pt. 2):677–684. Epub 2007/02/03. jphysiol.2007.128652 [pii] 10.1113/jphysiol.2007.128652 17272339 PMC2075554

[27] Alvarez R, de Andres J, Yubero P, Vinas O, Mampel T, Iglesias R, et al A novel regulatory pathway of brown fat thermogenesis. Retinoic acid is a transcriptional activator of the mitochondrial uncoupling protein gene. J Biol Chem. 1995;270(10):5666–5673. Epub 1995/03/10. . 7890689 10.1074/jbc.270.10.5666

[28] Barbera MJ, Schluter A, Pedraza N, Iglesias R, Villarroya F, Giralt M. Peroxisome proliferator-activated receptor alpha activates transcription of the brown fat uncoupling protein-1 gene. A link between regulation of the thermogenic and lipid oxidation pathways in the brown fat cell. J Biol Chem. 2001;276(2):1486–1493. Epub 2000/10/26. 10.1074/jbc.M006246200 M006246200 [pii]. . 11050084

[29] Mottillo EP, Bloch AE, Leff T, Granneman JG. Lipolytic products activate peroxisome proliferator-activated receptor (PPAR) alpha and delta in brown adipocytes to match fatty acid oxidation with supply. J Biol Chem. 2012;287(30):25038–25048. Epub 2012/06/12. 10.1074/jbc.M112.374041 M112.374041 [pii]. 22685301 PMC3408177

[30] Vega RB, Huss JM, Kelly DP. The coactivator PGC-1 cooperates with peroxisome proliferator-activated receptor alpha in transcriptional control of nuclear genes encoding mitochondrial fatty acid oxidation enzymes. Mol Cell Biol. 2000;20(5):1868–1876. . 10669761 10.1128/mcb.20.5.1868-1876.2000 PMC85369

[31] Yu XX, Lewin DA, Forrest W, Adams SH. Cold elicits the simultaneous induction of fatty acid synthesis and beta-oxidation in murine brown adipose tissue: prediction from differential gene expression and confirmation in vivo. FASEB J. 2002;16(2):155–168. Epub 2002/01/31. 10.1096/fj.01-0568com 16/2/155 [pii]. . 11818363

[32] Vallerand AL, Perusse F, Bukowiecki LJ. Stimulatory effects of cold exposure and cold acclimation on glucose uptake in rat peripheral tissues. Am J Physiol. 1990;259(5 Pt 2):R1043–1049. Epub 1990/11/01. . 2240264 10.1152/ajpregu.1990.259.5.R1043

[33] Marette A, Bukowiecki LJ. Noradrenaline Stimulates Glucose-Transport in Rat Brown Adipocytes by Activating Thermogenesis—Evidence That Fatty-Acid Activation of Mitochondrial Respiration Enhances Glucose-Transport. Biochemical Journal. 1991;277:119–124. ISI:A1991FV58400017. 1713031 10.1042/bj2770119 PMC1151199

[34] Trayhurn P. Fatty acid synthesis in mouse brown adipose tissue. The influence of environmental temperature on the proportion of whole-body fatty acid synthesis in brown adipose tissue and the liver. Biochim Biophys Acta. 1981;664(3):549–560. Epub 1981/06/23. . 7272321 10.1016/0005-2760(81)90132-6

[35] Ruas JL, White JP, Rao RR, Kleiner S, Brannan KT, Harrison BC, et al A PGC-1alpha isoform induced by resistance training regulates skeletal muscle hypertrophy. Cell. 2012;151(6):1319–1331. Epub 2012/12/12. 10.1016/j.cell.2012.10.050 S0092-8674(12)01363-3 [pii]. 23217713 PMC3520615

[36] Miura S, Kai Y, Kamei Y, Ezaki O. Isoform-specific increases in murine skeletal muscle peroxisome proliferator-activated receptor-gamma coactivator-1alpha (PGC-1alpha) mRNA in response to beta2-adrenergic receptor activation and exercise. Endocrinology. 2008;149(9):4527–4533. Epub 2008/05/31. en.2008-0466 [pii] 10.1210/en.2008-0466 . 18511502

[37] Muzzin P, Revelli JP, Kuhne F, Gocayne JD, McCombie WR, Venter JC, et al An adipose tissue-specific beta-adrenergic receptor. Molecular cloning and down-regulation in obesity. J Biol Chem. 1991;266(35):24053–24058. Epub 1991/12/15. . 1721063

[38] Granneman JG, Lahners KN, Chaudhry A. Molecular cloning and expression of the rat beta 3-adrenergic receptor. Mol Pharmacol. 1991;40(6):895–899. Epub 1991/12/01. . 1684635

[39] Mazzucotelli A, Viguerie N, Tiraby C, Annicotte JS, Mairal A, Klimcakova E, et al The transcriptional coactivator peroxisome proliferator activated receptor (PPAR)gamma coactivator-1 alpha and the nuclear receptor PPAR alpha control the expression of glycerol kinase and metabolism genes independently of PPAR gamma activation in human white adipocytes. Diabetes. 2007;56(10):2467–2475. Epub 2007/07/25. db06-1465 [pii] 10.2337/db06-1465 . 17646210

[40] Kleiner S, Mepani RJ, Laznik D, Ye L, Jurczak MJ, Jornayvaz FR, et al Development of insulin resistance in mice lacking PGC-1alpha in adipose tissues. Proc Natl Acad Sci U S A. 2012;109(24):9635–9640. Epub 2012/05/31. 10.1073/pnas.1207287109 1207287109 [pii]. 22645355 PMC3386123

[41] Fisher FM, Kleiner S, Douris N, Fox EC, Mepani RJ, Verdeguer F, et al FGF21 regulates PGC-1alpha and browning of white adipose tissues in adaptive thermogenesis. Genes Dev. 2012;26(3):271–281. Epub 2012/02/04. 10.1101/gad.177857.111 26/3/271 [pii]. 22302939 PMC3278894

